# Supplementary material for: The Systems Biology Research Tool: evolvable open-source software
Source: BMC Syst Biol. 2008 Jun 29;2:55. doi: 10.1186/1752-0509-2-55 (PMC2446383; doi:10.1186/1752-0509-2-55)
Supplement: Additional file 1 — SBRT Archive. An archive of the current version of the Systems Biology Research Tool. [file 1752-0509-2-55-S1.zip › sbrt-1.4.0/doc/developers_guide/api/sbrt/kernel/fba/Fluxome.html]

Fluxome


|  |  |  |
| --- | --- | --- |
| **Overview** \| **Package** \| **Class** \| **Use** \| **Tree** \| **Deprecated** \| **Index** \| **Help** | |  |
| **PREV CLASS**   **NEXT CLASS** | **FRAMES**    **NO FRAMES**     **All Classes** |  |
| SUMMARY: NESTED \| FIELD \| CONSTR \| METHOD | DETAIL: FIELD \| CONSTR \| METHOD |  |


---


## sbrt.kernel.fba Interface Fluxome

**All Known Subinterfaces:**: CatalyzedFluxome, IrrevRxnFluxome

**All Known Implementing Classes:**: IrrevRxnFluxomeV1

---

``` public interface Fluxome ```

This interface is used to represent fluxomes for use in Flux Balance Analysis.
A fluxome is a set of biochemical reactions whose rates (i.e. fluxes)
may be subject to constraint. A fluxome is composed of

|  |  |
| --- | --- |
| • chemical species | |
| • the chemical reactions in which those chemical species participate | |
| • the constraints on the fluxes of those reactions, and | |
| • the enzymes that catalyze those reactions | |

Two types of reactions exist in a fluxome:
internal reactions and exchange reactions.
Each internal reaction is assumed to be catalyzed by an enzyme.
Since a single enzyme may catalyze multiple reactions,
and since different enzymes may catalyze the same reaction, each reaction
requires a unique name to identifiy it. This class keeps track of
all of these relationships.

A flux constraint can be represented as α ≤ V ≤ β,
where α and β are the lower and upper flux bounds, respectively,
and V is the flux under consideration. For a particular type of fluxome,
the allowed flux constraints may have a defined range. That is to say,
the allowed values of α and β may differ between fluxomes
of different types or even between reactions of different type within
the same fluxome. For example, a popular type of fluxome uses the
following convention:
0 ≤ α ≤ ∞, α ≤ β ≤ ∞ for
internal reactions and
-∞ ≤ α ≤ ∞, α ≤ β ≤ ∞ for
exchange reactions.
This interface provides a way to maintain the integrity of these
definitions or conventions using `isValid(Interval, String, double)`.

**Author:**
:   This interface was written and documented by Jeremiah Wright
    while in the Wagner lab.

---

| **Method Summary** | |
| --- | --- |
| `int` | `addedConstraints()`             Returns the total number of constraints added to this fluxome. |
| `boolean` | `areRxnNames(java.util.Collection<java.lang.String> c)`             Indicates if every string in the provided collection is the name of a reaction contained in this fluxome. |
| `int` | `exchangeRxns()`             Returns the total number of exchange reactions contained in this fluxome. |
| `FluxConstraints` | `getConstraints()`             Returns the set of all constraints for fluxes and mathematical expressions of fluxes in this fluxome. |
| `java.util.Collection<java.util.Set<java.lang.String>>` | `getEquivRxnNames()`             Returns a collection of sets of the names of stoichiometrically equivalent reactions contained in this fluxome. |
| `java.util.Set<java.lang.String>` | `getEquivRxnNames(java.lang.String reactionName)`             Returns the set of names of reactions in this fluxome that are stoichiometrically equivalent to the provided reaction. |
| `java.util.Set<java.lang.String>` | `getExchangeRxnNames()`             Returns the set of names of all exchange reactions contained in this fluxome. |
| `java.util.Map<java.lang.String,? extends ExchangeRxn>` | `getExchangeRxns()`             Returns the set of all exchange reactions contained in this fluxome. |
| `Interval` | `getFluxConstraint(java.lang.String reactionName)`             Returns the flux constraint corresponding to the provided reaction. |
| `java.util.Set<java.lang.String>` | `getInternalRxnNames()`             Returns the set of names of all internal chemical reactions contained in this fluxome. |
| `java.util.Map<java.lang.String,? extends ChemRxn>` | `getInternalRxns()`             Returns the set of all internal chemical reactions contained in this fluxome. |
| `java.util.Set<java.lang.String>` | `getReverseRxnNames(java.lang.String reactionName)`             Returns the set of names of reactions in this fluxome that are stoichiometrically equivalent to the provided reaction in reverse. |
| `ChemRxn` | `getRxn(java.lang.String reactionName)`             Returns the chemical reaction in this fluxome with the provided name. |
| `Interval` | `getRxnExprConstraint(MathExpr<java.lang.String> rxnNameExpr)`             Returns the constraint corresponding to the provided mathematical expression of reaction names. |
| `java.util.Set<java.lang.String>` | `getRxnNames()`             Returns the set of names of all chemical reactions contained in this fluxome. |
| `java.util.Map<java.lang.String,ChemRxn>` | `getRxns()`             Returns the set of all chemical reactions contained in this fluxome. |
| `java.util.Map<java.lang.String,ChemRxn>` | `getRxns(java.util.Collection<java.lang.String> rxnNames)`             Returns the chemical reactions with the provided names. |
| `java.util.Set<java.lang.String>` | `getSpecies()`             Returns the set of names of all chemical species participating in reactions contained in this fluxome. |
| `SparseDoubleMatrix<java.lang.String,java.lang.String>` | `getStoichiometryMatrix()`             Returns the stoichiometry matrix of this fluxome. |
| `int` | `internalRxns()`             Returns the total number of internal reactions contained in this fluxome. |
| `boolean` | `isFluxVector(java.util.Map<java.lang.String,java.lang.Double> fluxVector, double tolerance)`             Indicates if the provided flux vector lies within the feasible space of this fluxome, given the provided tolerance value. |
| `boolean` | `isRxnName(java.lang.String s)`             Indicates if the provided string is the name of a reaction contained in this fluxome. |
| `boolean` | `isSpecies(java.lang.String s)`             Indicates if the provided string is the name of a chemical species contained in this fluxome. |
| `boolean` | `isValid(Interval c, java.lang.String rxnName, double tolerance)`             Indicates if the provided interval is a valid flux constraint for the reaction with the provided name. |
| `int` | `redundantRxns()`             Returns the total number of redundant reactions contained in this fluxome. |
| `Fluxome` | `replaceConstraints(FluxConstraints constraints, double constraintTolerance)`             Returns a copy of this fluxome, but with all constraints replaced by those provided. |
| `int` | `rxns()`             Returns the total number of chemical reactions contained in this fluxome. |
| `int` | `species()`             Returns the total number of chemical species contained in this fluxome. |

**Method Detail**

### getRxns

```
java.util.Map<java.lang.String,ChemRxn> getRxns()
```

:   Returns the set of all chemical reactions contained in this fluxome.
    This set is the union of internal and exchange reactions.

    :   **Returns:**: the set of all chemical reactions contained in this fluxome.

---


### getRxns

```
java.util.Map<java.lang.String,ChemRxn> getRxns(java.util.Collection<java.lang.String> rxnNames)
```

:   Returns the chemical reactions with the provided names.

    :   **Parameters:**: `rxnNames` - the names of the reactions to be returned. **Returns:**: reaction names mapped to their reaction.

---


### getInternalRxns

```
java.util.Map<java.lang.String,? extends ChemRxn> getInternalRxns()
```

:   Returns the set of all internal chemical reactions contained
    in this fluxome.

    :   **Returns:**: the set of all internal chemical reactions contained in this fluxome.

---


### getExchangeRxns

```
java.util.Map<java.lang.String,? extends ExchangeRxn> getExchangeRxns()
```

:   Returns the set of all exchange reactions contained in this fluxome.

    :   **Returns:**: the set of all exchange reactions contained in this fluxome.

---


### getRxn

```
ChemRxn getRxn(java.lang.String reactionName)
```

:   Returns the chemical reaction in this fluxome with the provided name.

    :   **Parameters:**: `reactionName` - the name of the chemical reaction to be returned. **Returns:**: the chemical reaction in this fluxome with the provided name.

---


### getRxnNames

```
java.util.Set<java.lang.String> getRxnNames()
```

:   Returns the set of names of all chemical reactions
    contained in this fluxome.

    :   **Returns:**: the set of names of all chemical reactions contained in this fluxome.

---


### getInternalRxnNames

```
java.util.Set<java.lang.String> getInternalRxnNames()
```

:   Returns the set of names of all internal chemical reactions
    contained in this fluxome.

    :   **Returns:**: the set of names of all internal chemical reactions contained in this fluxome.

---


### getExchangeRxnNames

```
java.util.Set<java.lang.String> getExchangeRxnNames()
```

:   Returns the set of names of all exchange reactions contained
    in this fluxome.

    :   **Returns:**: the set of names of all exchange reactions contained in this fluxome.

---


### getSpecies

```
java.util.Set<java.lang.String> getSpecies()
```

:   Returns the set of names of all chemical species participating
    in reactions contained in this fluxome.

    :   **Returns:**: the set of names of all chemical species participating in reactions contained in this fluxome.

---


### getFluxConstraint

```
Interval getFluxConstraint(java.lang.String reactionName)
```

:   Returns the flux constraint corresponding to the provided
    reaction.

    :   **Parameters:**: `reactionName` - the name of the reaction whose flux constraint is to be returned. **Returns:**: the flux constraint corresponding to the provided reaction.

---


### getRxnExprConstraint

```
Interval getRxnExprConstraint(MathExpr<java.lang.String> rxnNameExpr)
```

:   Returns the constraint corresponding to the provided
    mathematical expression of reaction names.

    :   **Parameters:**: `rxnNameExpr` - the mathematical expression of reaction names whose constraint is to be returned. **Returns:**: the constraint corresponding to the provided mathematical expression of reaction names.

---


### getConstraints

```
FluxConstraints getConstraints()
```

:   Returns the set of all constraints for fluxes and
    mathematical expressions of fluxes in this fluxome.

    :   **Returns:**: the set of all constraints for fluxes and mathematical expressions of fluxes in this fluxome.

---


### getStoichiometryMatrix

```
SparseDoubleMatrix<java.lang.String,java.lang.String> getStoichiometryMatrix()
```

:   Returns the stoichiometry matrix of this fluxome.

    :   **Returns:**: the stoichiometry matrix of this fluxome. Row variables in this matrix are chemical species names, and column variables are reaction names.

---


### getEquivRxnNames

```
java.util.Collection<java.util.Set<java.lang.String>> getEquivRxnNames()
```

:   Returns a collection of sets of the names of stoichiometrically
    equivalent reactions contained in this fluxome.

    :   **Returns:**: a collection of sets of the names of stoichiometrically equivalent reactions contained in this fluxome.

---


### getEquivRxnNames

```
java.util.Set<java.lang.String> getEquivRxnNames(java.lang.String reactionName)
```

:   Returns the set of names of reactions in this fluxome that are
    stoichiometrically equivalent to the provided reaction.

    :   **Parameters:**: `reactionName` - the name of the reaction for which the names of stoichiometrically equivalent reactions will be returned. **Returns:**: the set of names of reactions in this fluxome that are stoichiometrically equivalent to the provided reaction.

---


### getReverseRxnNames

```
java.util.Set<java.lang.String> getReverseRxnNames(java.lang.String reactionName)
```

:   Returns the set of names of reactions in this fluxome that are
    stoichiometrically equivalent to the provided reaction in reverse.

    :   **Parameters:**: `reactionName` - the name of the reaction for which the names of stoichiometrically equivalent reverse reactions will be returned. **Returns:**: the set of names of reactions in this fluxome that are stoichiometrically equivalent to the provided reaction in reverse.

---


### isRxnName

```
boolean isRxnName(java.lang.String s)
```

:   Indicates if the provided string is the name of a reaction
    contained in this fluxome.

    :   **Parameters:**: `s` - the string to test. **Returns:**: `true` if the string is a valid reaction name; `false` otherwise.

---


### areRxnNames

```
boolean areRxnNames(java.util.Collection<java.lang.String> c)
```

:   Indicates if every string in the provided collection is the name
    of a reaction contained in this fluxome.

    :   **Parameters:**: `c` - a collection of strings. **Returns:**: `true` if every string is a valid reaction name; `false` otherwise.

---


### isSpecies

```
boolean isSpecies(java.lang.String s)
```

:   Indicates if the provided string is the name of a chemical species
    contained in this fluxome.

    :   **Parameters:**: `s` - the string to test. **Returns:**: `true` if the string is a valid chemical species; `false` otherwise.

---


### isFluxVector

```
boolean isFluxVector(java.util.Map<java.lang.String,java.lang.Double> fluxVector,
                     double tolerance)
```

:   Indicates if the provided flux vector lies within the feasible space
    of this fluxome, given the provided tolerance value.

    :   **Parameters:**: `fluxVector` - reaction names mapped to flux values.: `tolerance` - the amount by which a computed value is allowed to violate a defined constraint. This should be small positive number, like 1E-9 for example. **Returns:**: `true` if the vector satisfies all constraints; `false` otherwise.

---


### isValid

```
boolean isValid(Interval c,
                java.lang.String rxnName,
                double tolerance)
```

:   Indicates if the provided interval is a valid
    flux constraint for the reaction with the provided
    name.

    :   **Parameters:**: `c` - the flux constraint to test.: `rxnName` - the name of the reaction.: `tolerance` - the allowed amount by which the provided constraint can violate its maximum defined interval. This maximum defined interval is implementation-dependent. **Returns:**: `true` if the constraint is valid; `false` otherwise. **Throws:**: `java.lang.IllegalArgumentException` - if the provided reaction name does not exist in this fluxome: `java.lang.NullPointerException` - if the provided constraint is `null`

---


### rxns

```
int rxns()
```

:   Returns the total number of chemical reactions contained
    in this fluxome.

    :   **Returns:**: the total number of chemical reactions contained in this fluxome.

---


### internalRxns

```
int internalRxns()
```

:   Returns the total number of internal reactions contained
    in this fluxome.

    :   **Returns:**: the total number of internal reactions contained in this fluxome.

---


### exchangeRxns

```
int exchangeRxns()
```

:   Returns the total number of exchange reactions contained
    in this fluxome.

    :   **Returns:**: the total number of exchange reactions contained in this fluxome.

---


### species

```
int species()
```

:   Returns the total number of chemical species contained in this fluxome.

    :   **Returns:**: the total number of chemical species contained in this fluxome.

---


### redundantRxns

```
int redundantRxns()
```

:   Returns the total number of redundant reactions contained
    in this fluxome.

    For any group of
    stoichiometrically reactions of size N, the number of
    redundant reactions within that group is N - 1.
    The total number of redundant reactions in a fluxome
    equals the sum of redundant reactions over each group
    of stoichiometrically equivalent reactions.

    :   **Returns:**: the total number of redundant reactions contained in this fluxome. **See Also:**: `getEquivRxnNames()`

---


### addedConstraints

```
int addedConstraints()
```

:   Returns the total number of constraints added to this fluxome.

    :   **Returns:**: the total number of constraints added to this fluxome. **See Also:**: `replaceConstraints(FluxConstraints, double)`

---


### replaceConstraints

```
Fluxome replaceConstraints(FluxConstraints constraints,
                           double constraintTolerance)
```

:   Returns a copy of this fluxome, but with all constraints
    replaced by those provided. If a flux constraint for a
    particular reaction was not provided, its default value
    will be used.

    :   **Parameters:**: `constraints` - the new set of flux constraints on the system.: `constraintTolerance` - the allowed amount by which constraints can violate their maximum defined intervals. These maximum defined intervals are implementation-dependent. **Returns:**: a copy of this fluxome, but with all constraints replaced by those provided.


---


|  |  |  |
| --- | --- | --- |
| **Overview** \| **Package** \| **Class** \| **Use** \| **Tree** \| **Deprecated** \| **Index** \| **Help** | |  |
| **PREV CLASS**   **NEXT CLASS** | **FRAMES**    **NO FRAMES**     **All Classes** |  |
| SUMMARY: NESTED \| FIELD \| CONSTR \| METHOD | DETAIL: FIELD \| CONSTR \| METHOD |  |


---
